# Systematic Review-Based Treatment Algorithm for the Multidisciplinary Treatment of Lung Cancer Bone Metastases †

**DOI:** 10.3390/cancers16244144

**Published:** 2024-12-12

**Authors:** Ah Reum Lim, Won Sup Yoon, Sunmin Park, Chai Hong Rim

**Affiliations:** 1 Department of Internal Medicine, Division of Oncology, Korea University Ansan Hospital, Korea University, Ansan-si 15355, Republic of Korea; ahreumlim@korea.ac.kr; 2 Department of Radiation Oncology, Korea University Ansan Hospital, Korea University, Ansan-si 15355, Republic of Korea; irionyws@korea.ac.kr (W.S.Y.); sunmini815@gmail.com (S.P.)

**Keywords:** lung cancer, bone metastases, NSCLC, treatment algorithm

## Abstract

**Background:** The prognosis for patients with lung cancer bone metastases has improved with the use of novel systemic agents. These patients might need surgery or radiotherapy to alleviate symptoms or maintain function. However, there is currently no disease specific algorithm to guide multidisciplinary decisions. **Methods**: The inclusion criteria encompassed studies with ≥10 patients offering multivariate analysis data on survival that were published after 2000 until September 2023. Clinical factors were categorized based on their characteristics and the pooled hazard ratios (HRs) for each category were calculated. A treatment algorithm was proposed based on clinical importance and the pooled HRs. **Results**: Fifteen studies involving 3759 patients with lung cancer bone metastases were included. The median survival ranged between 1.8–28.3 months (median: 12.4). Among the studies involving patients with EGFR+ or treated with TKIs, the median survival ranged between 19.5–28.3 months. The most reported significant factor was ECOG performance (nine studies) followed by chemotherapy use (six studies). In the pooled analyses, the pooled HR [95% confidence interval (CI)] of the EGFR status category was 2.109 (1.345–3.305); the ECOG performance category was 2.007 (1.536–2.622); the visceral metastases category was 2.060 (1.370–3.098); the bone metastases characteristics category (e.g., multiplicity, weight-bearing bone metastases) was 1.910 (1.443–2.527); the body weight category was 1.805 (1.334–2.442); the anti-absorbants category was 1.784 (1.448–2.196); the systemic treatment category was 1.695 (1.407–2.041); the skeletal-related event category was 1.616 (1.063–2.458); the smoking status category was 1.530 (1.306–1.793); the gender category was 1.482 (1.270–1.729); and the histology category was 1.450 (1.186–1.772). **Conclusions**: Oncological prognoses are influenced by various interrelated factors. Our treatment algorithm supports multidisciplinary strategies for managing NSCLC bone metastases, considering the complex factors influencing prognosis.

## Simple Summary

Bone metastases are a common complication in lung cancer patients, significantly affecting their quality of life and prognosis. Recent advances in systemic treatments have improved survival rates, but managing these patients remains complex due to the lack of disease-specific guidelines. This study reviewed survival factors in 3759 patients across 15 studies and developed a treatment algorithm to aid multidisciplinary decision-making. Key prognostic factors included EGFR mutation status, ECOG performance, and systemic therapy use. The proposed algorithm highlights the importance of personalized and collaborative care in managing lung cancer bone metastases effectively.

## 1. Introduction

Lung cancer remains a significant global health burden, with bone metastases occurring in a substantial proportion of patients, impacting prognosis and quality of life [1,2]. Despite advancements in systemic therapies, the management of lung cancer bone metastases presents a complex challenge requiring a multidisciplinary approach to optimize patient outcomes [3]. Currently, a lack of standardized treatment algorithms impedes the ability of clinicians to make informed, evidence-based decisions regarding the management of these patients.

Bone metastases often lead to pain, pathological fractures, and compression of the spinal cord, necessitating a range of interventions, including surgery, radiotherapy, or medical treatments [4,5,6]. Guidelines from organizations such as the National Comprehensive Cancer Network (NCCN) or the European Society for Medical Oncology (ESMO) primarily focus on bone metastases of breast and prostate cancers, while guidance for lung cancer bone metastases is comparatively less concentrated [7,8]. According to the guidelines from the ESMO clinical practice for solid cancers with bone metastases, recommendations emphasize a comprehensive approach to the investigation and management of patients with bone metastases. It is recommended that discussions on bone metastases/bone lesions be conducted within a multidisciplinary team, ensuring integration with all relevant therapeutic modalities [8].

In recent years, the landscape of lung cancer treatment has evolved with the advent of novel systemic modalities, such as tyrosine kinase inhibitors (TKIs) and immunotherapy, leading to improvements in overall survival rates [9,10,11]. Furthermore, recent studies have consistently reported that local treatments for metastatic lesions of lung cancer with a limited burden significantly improve oncological prognosis [12,13]. In addition to the use of novel agents and the metastatic burden, the course of patients with NSCLC bone metastases is affected by various factors. The survival of patients was shown to be affected by performance status [14,15]; weight loss and smoking history are also known to be adverse factors [16,17]. Skeletal events, such as fractures or cord compression [17,18] and interventions, such as anti-absorbents or radiotherapy, have also been significant factors [16,19]. Nevertheless, therapeutic guidance that comprehensively considers these clinical factors is lacking.

To address this gap in clinical guidance, we conducted a systematic review aimed at identifying key prognostic factors and developing a comprehensive treatment algorithm for patients with lung cancer bone metastases. Our methodology involved an analysis of studies published after 2000 until September 2023, encompassing data from over 3700 patients across fifteen studies.

## 2. Method

### 2.1. Eligible Criteria

We adhered to the PRISMA guidelines [20], AMSTAR guidelines [21], and consulted the Cochrane Handbook for Systemic Reviews, Version 6.2, for methodological guidance throughout the study. In this work, the study presented in [22] is expanded upon. The primary objective of this research was to identify the key clinical factors influencing the survival of patients with lung cancer bone metastases. The secondary aim was to propose a clinical flowchart for managing lung cancer patients with bone metastases, based on the major clinical factors investigated. To be eligible for inclusion, studies were required to meet the following criteria: (1) clinical studies with a minimum of 10 patients diagnosed with lung cancer bone metastases; (2) provision of data impacting survival in multivariate analyses; and (3) publication after 2000 until September 2023 (considering that older studies may present survival outcomes differing from recent trends).

### 2.2. Data Source and Collection Process

We systematically searched four databases, including PubMed, MEDLINE, EMBASE, and the Cochrane Library, as recommended by the Cochrane Handbook [23], up to 18 September 2023. For EMBASE, the following search terms were utilized: (nsclc:ab,ti OR ‘lung cancer’:ab,ti OR sclc: ab,ti) AND (‘bone metastasis’:ti OR ‘bone metastases’:ti OR osseous:ti) AND survival:ab,ti AND [2000–2023]/py AND (HR OR ‘hazard ratio):ab,ti. In other databases, we applied the above search terms moderately, considering the provided search methods of each database. Reference lists of included studies were also searched for possible missing studies. The complete search terms and strategy are outlined in Supplementary Note S1. We searched for all studies meeting the inclusion criteria, irrespective of language. Conference abstracts were included if they met the eligibility criteria. In cases where multiple studies originated from the same institution, we included all studies unless there was an overlap in patient pools. If an overlap occurred, we adopted the study with the larger number of patients; in cases where patient numbers were similar (<10% difference), we adopted the more recent study.

For data collection, we used a pre-standardized sheet including, as follows: (1) general information including the names of authors, publication title and year, study design, and conflicts of interest; (2) clinical information regarding patients’ age, histology, smoking status, performance status, the presence of visceral metastases, the presence of multiple bone metastases, and chemotherapy profile; and (3) clinical outcomes of interest, including median overall survival (OS), the 1-year OS percentile, and clinical factors affecting OS found to be of significance in multivariate analyses. Two independent reviewers performed the above processes; any disagreement was resolved by mutual discussion or a re-investigation of the literature.

### 2.3. Risk of Bias and Quality Assessment

In the preliminary literature review, the majority of the studies were non-randomized. To evaluate the quality of the included studies, we utilized the Newcastle–Ottawa scale [24]. Given that a substantial number of the studies were case series lacking a control group, those scoring 7 to 9 points were deemed to be high-quality, 5 to 6 points were considered medium-quality, and those with 4 points or fewer were categorized as low-quality studies. Consequently, our study received a low-quality rating. Following the Cochrane Handbook’s recommendation to exclude observational studies with a high risk of bias from meta-analyses, we excluded low-quality studies with the authors’ consent.

### 2.4. Statistics

The primary endpoint of this study is a pooled hazard ratio (HR); HRs with possible statistical significance (*p* value of < 0.1) were acquired from eligible studies. In our preliminary search of the literature, the majority of possibly eligible studies were non-randomized studies. Therefore, a random effects model was used to calculate the pooled HR, considering possible clinical heterogeneity and treatment details among studies regarding metastases treatments, and also by referencing the Cochrane Handbook, which states that a random effects model should be used to synthesize data from observational studies. Pooled analyses were performed according to subgroups, encompassing various clinical factors into several categories. Based on preliminary analyses of the literature, subjects of subgroups included, as follows: patients’ performance; gender; smoking status; cachexic status; bone metastases character; visceral metastases; the use of an anti-absorbant (e.g., denosumab); systemic treatment; EGFR status; skeletal-related event (SRE); and cancer histology. When a comparative analysis was performed on three or more groups, the largest comparative analysis value was included in the category. Serologic markers were not included in categorical pooled analyses due to the large heterogeneity across studies and the low clinical commonality among markers.

The heterogeneity assessment was performed using Cochran’s Q test [25] and I^2^ statistics [26]; I^2^ values of 25%, 50%, and 75% were regarded as low, moderate, and high heterogeneity, respectively. Publication bias was assessed when a subgroup included more than 10 studies, using a visual funnel plot analysis and quantitative Egger’s test [27]. If asymmetry was visible in the funnel plot analysis and the 2-tailed *p* value was less than 0.1, the correction value was calculated and referenced using Duval and Tweedie’s trim-and-fill method [28]. All statistical analyses were performed using Comprehensive Meta-Analysis Software, Version 4 (Biostat Inc., Englewood, NJ, USA).

### 2.5. Suggestion of a Treatment Algorithm

A treatment algorithm was proposed in the Discussion Section, considering the pooled effect size and factors with clinical significance. In addition, existing treatment guidelines or consensus for metastatic lung cancer were systematically searched and qualitatively referenced. The PUBMED and Medline were searched for up to 11 November 2023, with search term of ‘lung cancer’:exp,ti AND (‘bone metastasis’:exp,ti OR metastatic:ti) AND (guidelines:ti OR consensus:ti). Literature presenting treatment strategies for bone metastases from various cancers, not specifically lung cancer, was excluded. Search strategies are shown in Supplementary Note S2.

### 2.6. Protocol

This study is registered in PROSPERO (CRD42024500256).

## 3. Results

### 3.1. Study Selection and Characteristics

**Figure 1:**
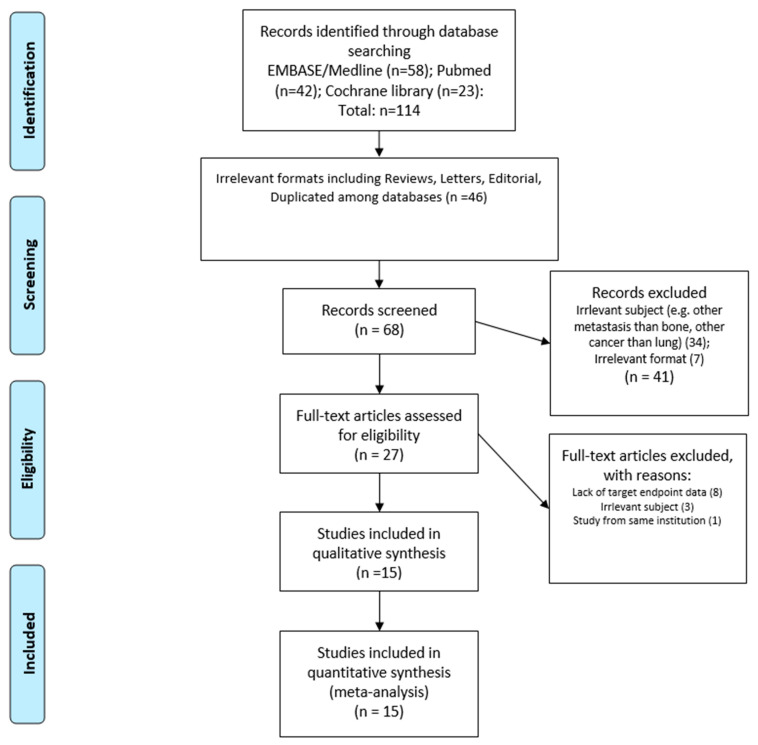
The study inclusion plot.

Among the total of 114 studies in the initial database search, 46 studies were removed due to duplicated studies among the databases, and 44 and 7 studies were excluded in the abstract screening due to irrelevant subject matter (e.g., metastases other than bone metastases, cancers other than lung cancer) or irrelevant formats such as reviews, letters, or editorials. Twenty-seven studies underwent a full-text review. Finally, 15 studies [14,15,16,17,18,19,29,30,31,32,33,34,35,36,37] involving 3759 lung cancer patients with bone metastases, fulfilling all the inclusion criteria, were included in the present systematic review. The study inclusion process is shown in Figure 1. Five studies were from China, three were from Japan, two were from France and Taiwan, and one was from Italy, Thailand, and Turkey. The earliest study recruited patients from 2000 to 2012, whereas the latest recruited patients from 2017 to 2020. Six studies reported no conflicts of interest; the remaining nine studies were supported by government or academic grants. No studies were commercially supported. General information about the studies are summarized in Table 1.

### 3.2. Quality Assessment

The representativeness of this analysis is high, as all studies focused on patients with a specific clinical category of lung cancer bone metastases. The ascertainment of exposure and assessment of outcomes were also met in all studies, as all information was obtained from secure medical records in tertiary hospitals. The main outcome of interest is the survival of patients with a specific disease (lung cancer bone metastases), which cannot be present before treatment in any of the studies. Therefore, the query regarding the absence of the outcome of interest at the study initiation was met in all studies. In this meta-analysis, clinical factors affecting survival were analyzed as the main endpoints. These factors were calculated retrospectively and statistically after estimating patient survival in all studies. Consequently, the studies included in this meta-analysis did not receive scores in queries regarding the comparability and selection of the non-exposed cohorts. Considering the short life expectancy of patients with lung cancer bone metastases, studies that were followed up for more than 1 year or had a difference of less than 20% between the follow-up period and median survival were scored for the query regarding a sufficient follow-up period. Regarding the query of the adequacy of follow-up, no study reported follow-up losses that could induce a significant bias. All studies were regarded as being of medium quality; therefore, all fifteen studies were involved in the pooled analyses. A detailed scoring sheet is provided in Supplement Table S1.

### 3.3. Clinical Results

**Figure 2:**
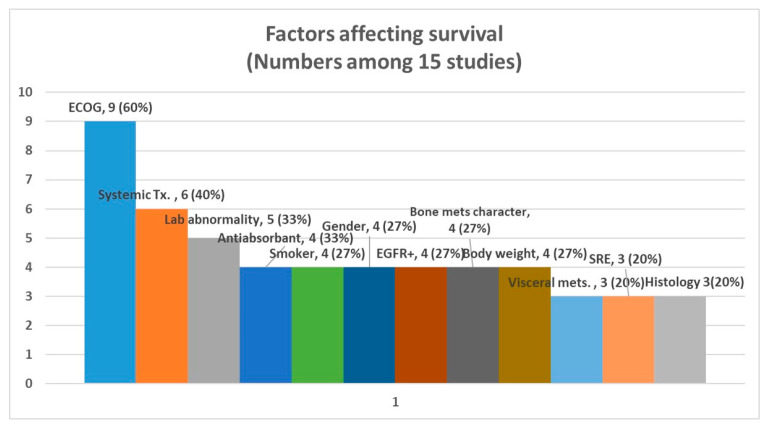
Significant clinical factors affecting survival among studies included.

The majority (14 out of 15) of the studies were retrospective. The median survival ranged from 1.8 to 28.3 months, with a median value of 12.4 months. Among the studies involving patients with EGFR+ status or treated with tyrosine kinase inhibitors, the median survival ranged from 19.5 to 28.3 months. In terms of clinical categories, the most commonly reported significant factor was ECOG performance status (9 out of 15 studies), followed by the use of systemic treatment (6 out of 15 studies) (Figure 2). Various serologic markers (lab abnormalities) were reported as significant in 5 out of 15 studies, although they were not included in the pooled analyses. A full list of factors with possible clinical significance in the multivariate analyses (*p* < 0.1) of the included studies is shown in Supplement Table S2.

**Figure 3:**
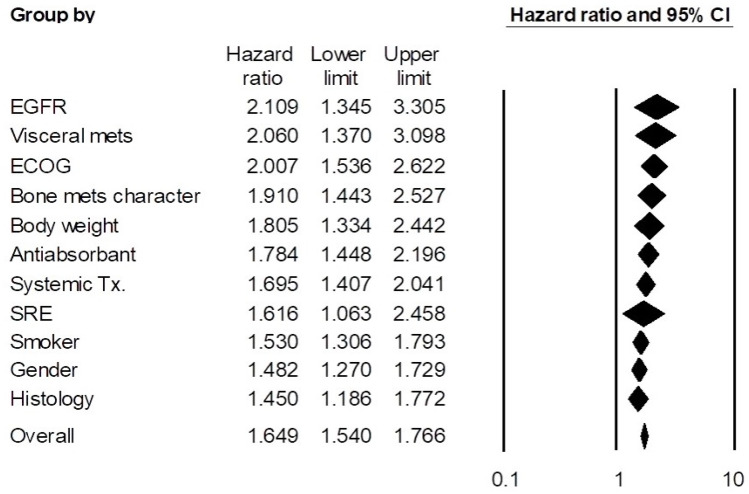
Forest plots of pooled analysis of significant factor categories.

In the pooled analyses, ordered by the size of pooled HRs, the pooled HR [95% confidence interval (CI)] of the EGFR status category was 2.109 (1.345–3.305); the ECOG performance category was 2.007 (1.536–2.622); the visceral metastases category was 2.060 (1.370–3.098); the bone metastases characteristics (e.g., multiplicity, weight-bearing bone metastases) category was 1.910 (1.443–2.527); the body weight category was 1.805 (1.334–2.442); the anti-absorbants category was 1.784 (1.448–2.196); the systemic treatment category was 1.695 (1.407–2.041); the skeletal-related events category was 1.616 (1.063–2.458); the smoking status category was 1.530 (1.306–1.793); the gender category was 1.482 (1.270–1.729); and the histology category was 1.450 (1.186–1.772). Heterogeneity was mostly moderate or less than moderate, although it was relatively high in pooled analyses of the EGFR status and ECOG performance categories (Table 2). Forest plots are shown in Figure 3. Publication bias assessment was not performed because none of the categories included more than 10 studies.

## 4. Discussion

### 4.1. Implication from the Results

In the past, the life expectancy of patients with lung cancer bone metastases was thought to be limited. However, as shown in this meta-analysis, patient life expectancy varies significantly, with some studies reporting median survival times exceeding 2 years [14,18,29]. Our objective in this meta-analysis was to identify the primary factors influencing the survival of patients with lung cancer bone metastases to help with clinical decisions. Performance status emerged as the most critical factor for treatment consideration. EGFR status demonstrated the highest pooled HR, particularly showcasing relatively high survival times among EGFR-positive patients across the included studies. Visceral metastases or metastatic characteristics were also identified as significant factors. Nevertheless, the pooled HR of all categories generally ranged between 1.5 and 2.1, and several clinical indicators were interconnected factors (e.g., performance status and body weight; EGFR status and systemic treatment). Hence, the principal implication is that multiple clinical factors influence the survival of lung cancer bone metastases patients, often working synergistically. Optimal treatment strategies appear to involve multidisciplinary treatment discussions and patient-tailored approaches that offer a range of modalities.

### 4.2. Role of Local Treatments

Oncological local modalities applicable to bone metastases of lung cancer encompass surgery and radiotherapy. Local modalities can reduce pain and decreased quality of life caused by bone metastases and have the potential to improve oncological prognosis for patients with bone metastases with limited burden. Although surgery and radiotherapy share some therapeutic roles for bone metastases, each presents distinct advantages and disadvantages. The primary advantage of surgical intervention lies in its capacity to promptly ameliorate symptoms in oncologic emergencies, such as fractures or neurological deficits and related pain [38,39]. In cases of severe neurological symptoms attributable to spinal metastases, immediate palliation can be achieved through surgeries such as laminectomy. Fixation procedures restore bone stability in instances of advanced fractures causing symptoms [40]. Conversely, radiotherapy is beneficial as it offers non-invasive and effective palliation, though it may require a few days to elicit a treatment response. For patients with comorbidities having a short life expectancy, radiotherapy plays a pivotal role in symptom alleviation without the physical burden associated with surgery [41]. In addition, a precise radiotherapy with high therapeutic efficiency called stereotactic radiosurgery has been increasingly used. Recent studies indicate that metastases-directed local treatments could improve survival and recurrence rates, particularly in lung cancer patients with a limited metastatic disease burden [12,13].

### 4.3. Role of Systemic Modalities

In recent years, the landscape of systemic therapy for advanced lung cancer has undergone significant advancements, with the emergence of novel anticancer drugs demonstrating efficacy in the treatment of metastatic disease [11]. Systemic therapy, including immunotherapy, targeted therapy, and chemotherapy, is fundamental to the treatment of advanced lung cancer. Targeted therapies have emerged as key components of systemic treatment for lung cancer bone metastases [9,10,42]. For patients with non-small cell lung cancer (NSCLC) harboring specific molecular alterations, such as epidermal growth factor receptor (EGFR) mutations or anaplastic lymphoma kinase (ALK) rearrangements, targeted therapies offer the promise of prolonged disease control and improved survival outcomes [43]. Agents, such as EGFR tyrosine kinase inhibitors (TKIs) and ALK inhibitors, have changed the treatment paradigm for patients with actionable mutations, providing a personalized approach to systemic therapy for lung cancer bone metastases [44]. Another significant advancement in the systemic management of lung cancer is the advent of immunotherapy [45]. Immune checkpoint inhibitors (ICIs), such as programmed cell death protein 1 (PD-1) and programmed death-ligand 1 (PD-L1) inhibitors, have demonstrated durable responses and improved survival in patients with advanced NSCLC [46]. These agents harness the body’s immune system to target cancer cells, offering a potential therapeutic option for patients with bone metastases who may have limited treatment alternatives [47]. In addition, platinum-containing doublet chemotherapy is the standard treatment of driver-gene-negative NSCLC, which has proven to be superior to monotherapy in overall survival [48].

In patients with NSCLC who have bone metastases, bone-modifying agents such as denosumab and bisphosphonates are recommended [49]. Bone-modifying agent therapy can decrease bone complications, such as pain reduction or delayed skeletal events [50,51]. The FDA has approved the use of denosumab and zoledronic acid in patients with bone metastases from solid tumors [52].

### 4.4. Clinical Workflow Suggestion

In our search for guidelines or a consensus regarding the treatment of bone metastases in lung cancer, we identified six results from various consortiums [41,53,54,55,56]. Among these, the consensus from the Chinese experts’ consortium stands out as the only report solely focused on the treatment of bone metastases in lung cancer [56]. Their findings suggest that multidisciplinary treatment is essential due to the diverse nature of bone metastases and the range of treatment options available. The guidelines from the American College of Chest Physicians [41] provide indications for surgical treatment (involving >50% of the cortex, expected survival > 4 weeks, solitary well-defined lytic lesions), as well as an analysis of the efficacies of short-term and conventional radiotherapy. They discuss the utility of bisphosphonates and radiopharmaceutical agents as well, highlighting the efficacy of various treatments. Other guidelines delve into medical treatments, such as chemotherapy and bisphosphonates, in detail, but offer limited information on the effectiveness of local modalities. Although Coleman et al. [57]. and the ESMO group [8] reported a practical clinical flow that considered disease burden, life expectancy, and the effective indication of anti-absorbents, they included lung cancer and other cancers in the analysis (reason for exclusion) and were not based on statistical clinical factors. Overall, existing guidelines or consensuses have limitations in serving as clinical decision-making tools for treating patients with lung cancer bone metastases.

**Figure 4:**
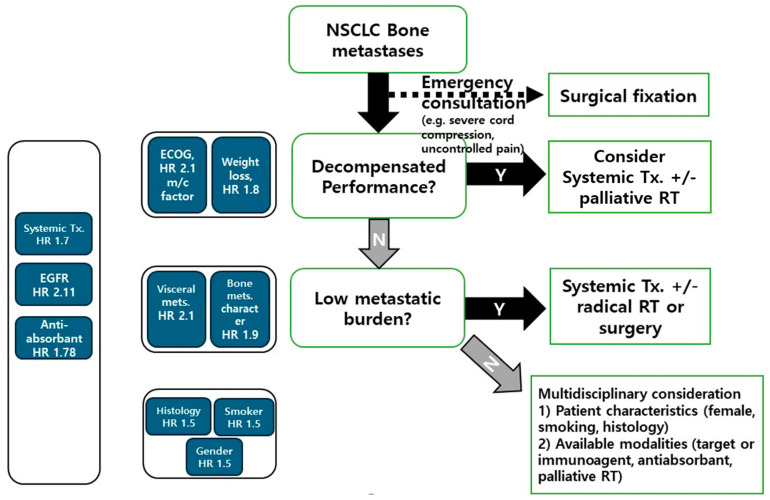
Suggested multidisciplinary algorithm for bone metastases treatment. NSCLC, non-small cell lung cancer, RT, radiotherapy, ECOG, Eastern Cooperative Group, HR, hazard ratio, Tx, treatment. The dotted arrow indicates an optional progression.

In the actual field of treating patients with lung cancer bone metastases, professionals from various disciplines convene to discuss treatment approaches. However, the absence of a standardized treatment algorithm complicates decision making. Furthermore, most related studies are retrospective in design and utilize diverse treatment modalities; therefore, deriving practically useful information is difficult. To address this challenge, we propose a treatment algorithm based on the findings of this meta-analysis (Figure 4).

This algorithm considers clinical factors with relatively high pooled HRs and the practical clinical situations of NSCLC bone metastases. The use of systemic treatments, anti-absorbants, and EGFR status, all of which are correlated, emerged as significant prognostic factors. Given their inclusion within the broader category of systemic treatment, the algorithm considers systemic treatment as the mainstay modality for patients with bone metastases. The first clinical category considered in therapeutic decisions is performance status. This category is based on the ECOG performance status, which is the most commonly reported factor and has a high pooled HR, and weight loss, which is a clinical factor that can affect, and is correlated with, performance. Palliative local treatment, such as palliative radiotherapy, can be considered for patients with a decompensated performance status. Furthermore, the algorithm accounts for metastatic burden, which can encompass the categories of visceral metastases and bone metastases characteristics, both of which have shown high pooled HRs. Recent evidence suggests that active local treatment may enhance survival and progression-free rates in lung cancer metastatic patients with low metastatic burdens [12,13]. Consequently, our algorithm advocates for considering radical radiotherapy (i.e., stereotactic radiotherapy with ablative dose of radiation) or surgery alongside systemic treatment in such scenarios. In addition, the treatment decision may consider emergency consultations that necessitate surgical fixation. Oncologic emergencies encompass conditions that can cause irreversible dysfunction within hours to days, potentially resulting in early mortality, including, for example, severe spinal cord compression or uncontrolled pain [58]. While emergency situations have rarely been studied as a statistical factor, there is little clinical disagreement considering the necessity of immediate palliation through surgical intervention [59]. After considering the above, treatment methods should be selected through a multidisciplinary approach, considering various clinical factors and the options available at healthcare institutions. While this algorithm has limitations in providing detailed information on specific treatments, it can serve as a helpful tool for guiding clinical decisions.

### 4.5. Limitations

The number of factors is too large to analyze each factor for all the studies included in our meta-analysis; analyzing all factors separately has little clinical utility. Therefore, we categorized various factors for analysis; this method has the advantage of providing a broad understanding of the literature at a glance, but it also has the limitation of lacking detailed insights into the characteristics of each factor. In addition, the proposed clinical algorithm is based on meta-analysis results but has been guided by expert opinions and could require validation through independent patient data. Therefore, our results and algorithm should not be considered as robust evidence but rather as a possible reference that can be used for treatment decisions. Additionally, our meta-analysis results did not identify one or two dominant factors, suggesting that several factors have a complex effect on prognosis. Therefore, the algorithm we propose can serve as an example; based on the pooled analysis results, a treatment strategy tailored to the local situation may be designed.

### 4.6. Areas of Improvement

Lung cancer bone metastases is a disease with a wide range of aspects and prognoses. Furthermore, the prognosis for patients has improved thanks to novel agents and advancements in local treatment methods. Therefore, various treatments are used together for lung cancer bone metastases. However, most studies on lung cancer bone metastases tend to be analyzed from the perspective of a specific researcher. Therefore, actual clinical decisions are made based on integrated insight and clinical experience from various studies. This study serves to strengthen such integrated insights and provides data that can be referred to when creating guidelines containing details of future treatments.

## 5. Conclusions

The oncological prognosis of lung cancer bone metastases is influenced by various factors that often interact with each other. Given the multifaceted nature of factors and therapeutic approaches affecting the patient’s prognosis, our treatment algorithm could help in the design of multidisciplinary treatment strategies for NSCLC bone metastases.

## Figures and Tables

**Table 1:** Overall characteristics of included studies.

Author, Publication Year, Country	Patient Recruit	Study Design	No. Patients	Specific Disease Entity	Age	Adeno/Sqcc/Others (%)	Smoker (%)	ECOG ≥2 (%)	Multiple Bone Mets (%)	Visceral Mets (%)	CTx Profile	MOS (Months)	CoI of Funding Sources
Lagana, 2022 Italian [13]	2014–2019	R	247	NSCLC, EGFR mutated	50	all adenocarcinoma	66%	9%	1–2: 47% 3–4: 31% 5–10: 22%	96	Gefitinib: 67% Erlotinib: 16% Afatinib: 17% Osimertinib: 0.4%	28	None
Ulas, 2018 Turkey [14]	2000–2012	R	335	NSCLC	58	50.4/49.6%	76.4%	54%	57.9%		Cisplatin/gemcitabine: 26.9% Cisplatin/etoposide:12.8% Cisplatin/vinorelbine: 9.0% Cisplatin/docetaxel: 8.1% Carboplatin/vinorelbine: 6.0%	12 (*w*/*o* SRE) vs. 7 (SRE)	None
Ko, 2022 Taiwan [17]	2016–2018	R	190	NSCLC, EGFR mutated	none	94.2/5.8%	84.2%	21%	82.6%	Lung/pleura: 69.5% Liver: 17.4% Adrenal/renal: 15.8% Abdominal LNs: 8.4%	Gefitinib, Erlotinib: 36.8% Afatinib: 63.1%	26.6 (denosumab) vs. 20.1 (no denosumab)	Government, academic
Yap, 2019 Taiwan [15]	2000–2013	R	477	Lung cancer receiving bone RTx	mean age 62.86	55.8/11.1/33.1	49.9%	35.2%		14.5		5.2	Government, academic
Pruksakorn, 2018 Thailand [29]	2006–2013	R	505	Lung cancer	none	61.4/25.9/6.5%		27.9%	70.3%	29.9%	Cisplatin based CTx.	4.9	Academic
Cui X, 2019 China [28]	2005–2017	R	129	NSCLC, TKI-treated	55.9	89.1/10.9	58.9%	11.6%	79.8%	Brain: 24.8%	CTx.: 52.7% EGFR TKI: 33.3% ALK TKI:4.7%	28.3 (combination) vs. 22.0	Government
Xu S, 2021 China [33]	2008–2010	R	234	NSCLC	57	70.9/27.4/1.7	45.7%	30.8%	82.5%	40.2%		8.9	Government
Chambard, 2018 France [18]	2011-	P	64	NSCLC	65	100/0/0	85%	39%	87%	Lung: 25% Liver: 30% Brain: 19% Adrenal: 31%	paclitaxel/carboplatin: 58% pemetrexed/cisplatin: 20% EGFR inhibitor: 13%	7	Academic
Deberne, 2014 France [16]	2003–2010	R	55	NSCLC	62.5	78.2/3.6/18.2		38.1%	81.8%	54.5%		8.15	None
Udagawa, 2017 Japan [32]	2010–2014	R	149	NSCLC	none	91.2/8.7	66.4%	16.8%	77.9%	64.4%	Platinum based CTx: 59.1% EGFR TKI: 40.3%	21.4 (denosumab) vs. 12.7 (zoledronic acid) vs. 10.5 (No Tx)	None
Qiang, H, 2022 China [30]	2017–2020	R	110	NSCLC pembrolizumab-treated	none	56.3/27.3/16.4	60.0%	5.5%		32.7%	Pembro mono: 34.5% Pembro combination: 65.5%	14.8	Academic
Zhang G, 2017 China [35]	2012–2015	R	552	NSCLC, EGFR mutated	mean age 60	88.2/11.8%	28.3%	8.7%		37.1%	CTx.: 49.5% TKI: 37.1%	20.5 (biosphosphonate) vs. 19.5 (no biosphosphonate)	Government
Sunaga, 2017 Japan [36]	2006–2014	R	98	Lung cancer Zoledronic acid-treated	70	80.6/19.4	n				CTx.: 50% Non-CTx.: 50%	109 (fever > 37) vs. 55 (no fever < 37)	None
Dohzono, 2020 Japan [31]	2009–2017	R	198	Lung cancer	mean age 68.9	88/12%		36%	62%	46%	EGFR TKI: 26%	21.4 (EGFR TKI) vs. 6.1 (no TKI, NSCLC) vs. 4.9 (SCLC)	None

Abbreviations: ECOG, Eastern Cooperative Oncology Group performance status; CTx, chemotherapy; MOS, median overall survival; NSCLC, non-small cell lung cancer; R, retrospective; P, prospective; SRE, skeletal-relative events; RTx, radiotherapy; TKI, tyrosine kinase inhibitor; SCLC, small cell lung cancer, CoI, conflicts of interests.

**Table 2:** Pooled results of clinical categories.

Category	No of Pooled Studies	Heterogeneity p, I^2^%	Pooled HR (95% CI)
EGFR	4	<0.001, 87.4%	2.109 (1.345–3.305)
ECOG	9	0.001, 69.5%	2.007 (1.536–2.622)
Visceral mets	3	<0.001, 50.1%	2.060 (1.370–3.098)
Bone mets character	4	0.213, 33.2%	1.910 (1.443–2.527)
Body weight	4	0.084, 54.8%	1.805 (1.334–2.442)
Anti-absorbant	4	0.553, ~0%	1.784 (1.448–2.196)
Systemic Tx.	5	0.365, 7.3%	1.695 (1.407–2.041)
SRE	3	0.129, 51.1%	1.616 (1.063–2.458)
Smoker	3	0.915, ~0.0%	1.530 (1.306–1.793)
Gender	4	0.351, 8.4%	1.482 (1.270–1.729)
Histology	3	0.609, ~0.0%	1.450 (1.186–1.772)

Abbreviations: HR, hazard ratio; CI, confidence interval; ECOG, Eastern Cooperative Group; EGFR, epidermal growth factor receptor; mets, metastases; SRE, skeletal-related event; Tx., treatment.

## Data Availability

The data presented in this study are available in this article and Supplementary Materials.

## Supplementary Materials

The following supporting information can be downloaded at: https://www.mdpi.com/article/10.3390/cancers16244144/s1, Note S1: Search terms for main analyses; Note S2: Draft of search strategy of existing guidelines references; Table S1: Scoring sheet according to New-Castle Ottawa scale; Table S2: List of factors with *p* value of <0.1 in multivariate analyses, included in pooled categorical analyses (except serologic markers).
