# Supplementary material for: Systematic Review-Based Treatment Algorithm for the Multidisciplinary Treatment of Lung Cancer Bone Metastases
Source: Cancers (Basel). 2024 Dec 12;16(24):4144. doi: 10.3390/cancers16244144 (PMC11674356; doi:10.3390/cancers16244144)
Supplement: Supplementary file 1 [file cancers-16-04144-s001.zip › cancers-3320896-supplementary.pdf]

Supplementary Note S1. Search terms for main analyses

Search term in Embase and Medline

(nsclc:ab,ti OR 'lung cancer':ab,ti OR sclc:ab,ti) AND ('bone metastasis':ti OR 'bone metastases':ti OR osseous:ti) AND survival:ab,ti AND [2000-2023]/py AND (HR OR 'hazard ratio):ab,ti

Search term in Pubmed

(nsclc[Title/Abstract] OR 'lung cancer'[Title/Abstract] OR sclc[Title/Abstract]) AND ('bone metastasis'[Title] OR 'bone metastases'[Title] OR osseous[Title]) AND survival[Title/Abstract] AND (HR[Title/Abstract] OR 'hazard ratio[Title/Abstract])

Search term in Cochrane library

(nsclc OR "lung cancer" OR sclc) AND ("bone metastasis" OR "bone metastases" OR osseous) AND survival AND (HR OR "hazard ratio")

Supplement Note S2. Draft of search strategy of existing guidelines references

Searched for PUBMED and Medline, up to 11th November 2023

Search term: 'lung cancer':exp,ti AND ('bone metastasis':exp,ti OR metastatic:ti) AND (guidelines:ti OR consensus:ti)
Inclusion criteria: Consensus or guidelines suggesting treatment strategies for bone metastasis of lung cancer

Exclusion criteria: Literature that comprehensively analyzed various cancers other than lung cancer was excluded.

Among 122 searched studies found, six studies (in below table) those are guidelines or consensus for lung cancer treatment of metastatic or bone metastasis or advanced (palliative care) disease are qualitatively analyzed.

**Title**	**Affiliation**	**Year**	**Disease specificity**	**Cancer type**
Chinese expert consensus on the diagnosis and treatment of bone metastasis in lung cancer (2022 edition)	Chinese Expert Consensus	2022	Bone mets	Lung cancer
Metastatic non-small cell lung cancer: ESMO Clinical Practice Guidelines for diagnosis, treatment and follow-up	European Society of Medical Oncology	2019	Metastatic disease	Lung cancer
The Japanese Lung Cancer Society Guideline for non-small cell lung cancer, stage IV	Japanese Lung Cancer Society	2019	Metastatic disease	Lung cancer
Treatment of non-small cell lung cancer: Advanced (metastatic) disease. Guidelines of clinical practice made by the European Lung Cancer Working Party	European Lung Cancer Working Party	2007	Metastatic disease	Lung cancer
Palliative care in lung cancer: ACCP evidence-based clinical practice guidelines (2nd edition)	American College of Chest Physician	2007	Advanced disease	Lung cancer
Treatment of metastatic non-small cell lung cancer: 2018 guidelines of the Italian Association of Medical Oncology (AIOM)	Italian Association of Medical Oncology	2019	Metastatic disease	Lung cancer

Supplement Table S1. Scoring sheet according to New-Castle Ottawa scale.

Query No	Selection				Comparability	Outcome			**Overall score (9 to be full)**
	1	2	3	4	5	6	7	8	
	Representativeness of the exposed cohort	Selection of the non exposed cohort	Ascertainment of exposure	Outcome of interest was not present at start of study	Comparability of cohorts on the basis of the design or analysis	Assessment of outcome	Was follow-up long enough for outcomes to occur	Adequacy of follow up of cohorts	
Lagana	1	0	1	1	0	1	1	1	6
Ulas	1	0	1	1	0	1	1	1	6
Ko	1	0	1	1	0	1	0	1	5
Yap	1	0	1	1	0	1	1	1	6
Pruksakorn	1	0	1	1	0	1	0	1	5
Cui X	1	0	1	1	0	1	0	1	5
Xu S	1	0	1	1	0	1	0	1	5
Chambard	1	0	1	1	0	1	1	1	6
Deberne	1	0	1	1	0	1	1	1	6
Udagawa	1	0	1	1	0	1	1	1	6
Qiang, H	1	0	1	1	0	1	0	1	5
Zhang G	1	0	1	1	0	1	0	1	5
Sunaga	1	0	1	1	0	1	0	1	5
Dohzono	1	0	1	1	0	1	0	1	5
Hui	1	0	1	1	0	1	0	1	5

Supplement Table S2. List of factors with *p* value of <0.1 in multivariate analyses, included in pooled categorical analyses (except serologic markers).

References, publication year, country	Factors significant in MVA	References, publication year, country	Factors significant in MVA
Lagana, 2022 Italian	Smoker: 1.635 (1.134–2.356, 0.008)<br>ECOG 2-4: 2.360 (1.296-4.297, 0.005)<br>Synchronous mets: 0.481 (0.328-0.707, 0.000)	Chambard, 2018 France	DKK1 elevation: 1.31 (1.00-1.71, 0.05)<br>HbA1C decreased: 2.08 (1.20-3.57, 0.009)<br>ECOG 2-4: 5.76 (1.61-20.64, 0.007)<br>Antiresorptive drugs: 0.22 (0.05-0.87, 0.031)
Ulas, 2018 Turkey	Female: 0.56 (0.40-0.79, 0.001)<br>ECOG 2-4: 1.53 (1.12-2.10, 0.008)<br>High LDH level: 0.55 (0.38-0.80, 0.002)<br>High Calcium level: 0.56 (0.31-1.03, 0.06)<br>Hypoalbuminemia: 0.67 (0.49-0.92, 0.01)<br>Bone mets at Dx: 2.10 (1.39-3.18, <0.001)<br>Multiple bone mets: 1.50 (1.08-2.10, 0.01)<br>the presence of SREs: 1.19 (0.80-1.76, 0.03)<br>the presence of Bisphosphonate Tx: 0.60 (0.43-0.83, 0.003)<br>the presence of Palliative RT: 0.51 (0.34-0.77, 0.001)	Deberne, 2014 France	T stage (T0-1 vs. 2-4): 2.8 (0.004)<br>Weight loss >10%: 3.1 (0.02)<br>TTF 1 negative: 2.42 (0.004)<br>Spinal epidural mets: 2.5 (0.0036)<br>CRP: 4.3 (0.002)
Ko, 2022 Taiwan	ECOG 2-4: 1.932 (1.261-2.959, 0.002)<br>adrenal/renal mets: 2.914 (1.825-4.654, <0.0011)<br>1st line Afatinib: 0.567 (0.387-0.832, 0.004)<br>SRE at Dx: 1.659 (0.971-2.831, 0.064)<br>Denosumab use: 0.594 (0.408-0.865, 0.007)	Udagawa, 2017 Japan	Denosumab: 0.513 (0.342-0.757, <0.01)<br>Smoking status: 0.653 (0.428-0.957, 0.03)<br>EGFR Del/L858R: 0.378 (0.256-0.557, <0.01)<br>Visceral mets: 0.466 (0.311-0.689, <0.01)
Yap, 2019 Taiwan	Age (<0.001)<br>BMI, <18.5 (vs. >25): 2.31 (1.56-3.44, <0.001)<br>NSCLC (vs. SCLC): 0.59 (0.41-0.86, 0.01)<br>EGFR positive (vs. unknown): 0.66 (0.46-0.93, 0.02)<br>Smoker: 1.50 (1.24-1.83, <0.001)	Qiang, H, 2022 China	ECOG 0-1: 0.117 (0.040-0.345, <0.0001)<br>Treatment line (1 vs. ≥2): 0.372 (0.190-0.728, 0.004)
Pruksakorn, 2018 Thailand	Male: 1.42 (1.166-1.729, <0.001)<br>ECOG 3-4: 1.30 (1.056-1.591, 0.013)<br>NSCLC (vs. SCLC): 1.42 (0.945-2.131, 0.091)	Zhang G, 2017 China	ECOG 0-1: 0.573 (0.192-0.961, 0.038)<br>EGFR mutation: 0.710 (0.597-0.913, 0.021)
Cui X, 2019 China	Female: 0.32 (0.09-1.03, 0.057)<br>Weight-bearing bone: 6.29 (1.27-31.1, 0.024)	Sunaga, 2017 Japan	High WBC count:2.834 (1.645-4.883, <0.001)<br>High LDH: 3.044 (1.831-5.060, <0.001)<br>Fever (>37): 0.603 (0.372-0.976, 0.039)<br>Chemo (administered): 0.481 (0.285-0.811, 0.006)<br>Hypercalcemia: 2.453 (1.422-4.232, 0.001)
Xu S, 2021 China	Male: 1.349 (1.029-1.768, 0.030)<br>Nonadeno: 1.334 (0.993-1.793, 0.056)<br>ECOG ≥2: 1.674 (1.256-2.232, <0.001)<br>SII(systemic immune-inflammation index): 1.456 (1.100-1.927,	Dohzono, 2020 Japan	Psoas major area <25%: 1.39 (0.79-1.75, 0.092)<br>Paravertebral muscles area <25%: 1.73 (1.17-2.56, 0.006)<br>EGFR Tx: 0.26 (0.17-0.40, <0.001)<br>Abnormal lab data: 1.46 (0.96-2.24, 0.079)<br>Visceral or cerebral mets: 1.35 (0.99-2.24, 0.057)

	0.009)<br>Systemic CTx: 0.596 (0.437-0.813, 0.001)		ECOG 3-4: 2.60 (0.82-3.70, <0.001)<br>Previous CTx: 1.34 (0.96-1.88, 0.088)

MVA, multivariate analyses; ECOG, Eastern Cooperative Oncology Group performance scale; SRE, skeletal related events; Dx, diagnosis; BMI, body mass index, CTx, chemotherapy
